# Spatial mapping of photovoltage and light-induced displacement of on-chip coupled piezo/photodiodes by Kelvin probe force microscopy under modulated illumination

**DOI:** 10.3762/bjnano.14.87

**Published:** 2023-11-06

**Authors:** Zeinab Eftekhari, Nasim Rezaei, Hidde Stokkel, Jian-Yao Zheng, Andrea Cerreta, Ilka Hermes, Minh Nguyen, Guus Rijnders, Rebecca Saive

**Affiliations:** 1 Inorganic Materials Science, MESA+, University of Twente, Enschede, 7522NB, the Netherlands; 2 Park Systems Europe GmbH, 68199 Mannheim, Germany

**Keywords:** Kelvin probe force microscopy (KPFM), light-driven micro/nano systems, piezoelectric membrane, surface photovoltage (SPV), time-dependent AFM

## Abstract

In this work, a silicon photodiode integrated with a piezoelectric membrane is studied by Kelvin probe force microscopy (KPFM) under modulated illumination. Time-dependent KPFM enables simultaneous quantification of the surface photovoltage generated by the photodiode as well as the resulting mechanical oscillation of the piezoelectric membrane with vertical atomic resolution in real-time. This technique offers the opportunity to measure concurrently the optoelectronic and mechanical response of the device at the nanoscale. Furthermore, time-dependent atomic force microscopy (AFM) was employed to spatially map voltage-induced oscillation of various sizes of piezoelectric membranes without the photodiode to investigate their position- and size-dependent displacement.

## Introduction

Light has been recognized as a versatile external energy source to actuate micro/nanorobots with outstanding merits of wireless, remote, and precise controllability [1–4]. Light-driven micro/nanorobots convert light into mechanical motion and are able to perform precise motion with high resolution. This offers promising possibilities for biomedical, environmental, and micro/nanoengineering applications [5–6]. Various types of design and actuation mechanisms have been developed in recent years [7–8]. A primary requirement to unlock the better performance of these micro/nano devices is to scrutinize their structure and the interaction between their different components. This can be done by high-resolution characterization techniques that simultaneously probe dynamic properties of different parts of the device. This enables the decoupling of the roles of each function of the components on the overall motion behavior.

A variety of characterization techniques, such as white light interferometry, laser Doppler vibrometry (LDV), and double-beam laser interferometry (DBLI) have been used to determine the displacement of piezoelectric membranes [9–10]. However, the working principle of these techniques is based on optical interferometry mapping which can be challenging for light-sensitive devices. Furthermore, it can be advantageous to employ a method that also allows for mechanical contact and manipulation. Atomic force microscopy (AFM) [11–14] is a powerful and versatile technique to study fundamental and functional characteristics of materials and devices at the nanoscale, with application in physics, materials science, engineering, and biology. It can operate in either static (contact mode) or dynamic (tapping and noncontact mode) modes with atomic vertical resolution. In several studies, AFM has been used to determine photo-induced height/topography variation in organic–inorganic lead halide perovskites [15], nanosheets [16], and photosensitive polymers [17].

Kelvin probe force microscopy (KPFM), an electrostatic variant of AFM, can be used to measure contact potential difference (CPD) between the tip and the sample [18–20]. In particular, time-dependent KPFM [21–23] allows us to determine temporal changes of CPD and understand the dynamic behavior of functional devices at the nanoscale. Kelvin probe force microscopy in combination with illumination has been used to investigate photo-generated charge carriers of photovoltaic materials and devices. This is done by determining the CPD shift under illumination known as surface photovoltage (SPV) by calculating SPV = CPD_light_ − CPD_dark_, whereas CPD_dark_ is the CPD in the dark and CPD_light_ is the CPD under illumination in that same location [24–27]. In some studies, KPFM has been employed for simultaneous study of structural and optoelectronic properties of materials and functional devices [28–30]. For example, the topography and SPV of illuminated photostrictive materials have simultaneously been examined through KPFM to determine the generated photovoltage and the following lattice change of the material [31].

Here, we present a KPFM study involving modulated illumination to investigate local height changes (vertical displacement) and the correlative SPV of the light-driven piezo/photodiode device over time. Employing modulated illumination enabled us to quantify precisely the device displacement due to improved signal-to-noise ratio, which could not be achieved by continuous illumination. The configuration of the hybrid piezo/photodiode device has previously been developed and reported by our group [32].

In further investigations, we used time-dependent AFM to determine the voltage-induced displacement of solely piezoelectric membranes without the photodiode. This experiment was performed to probe the local electromechanical properties of the piezoelectric membrane as a reference sample, which can reveal its contribution to the piezo/photodiode device.

## Methodology

### Sample fabrication

**Figure 1:**
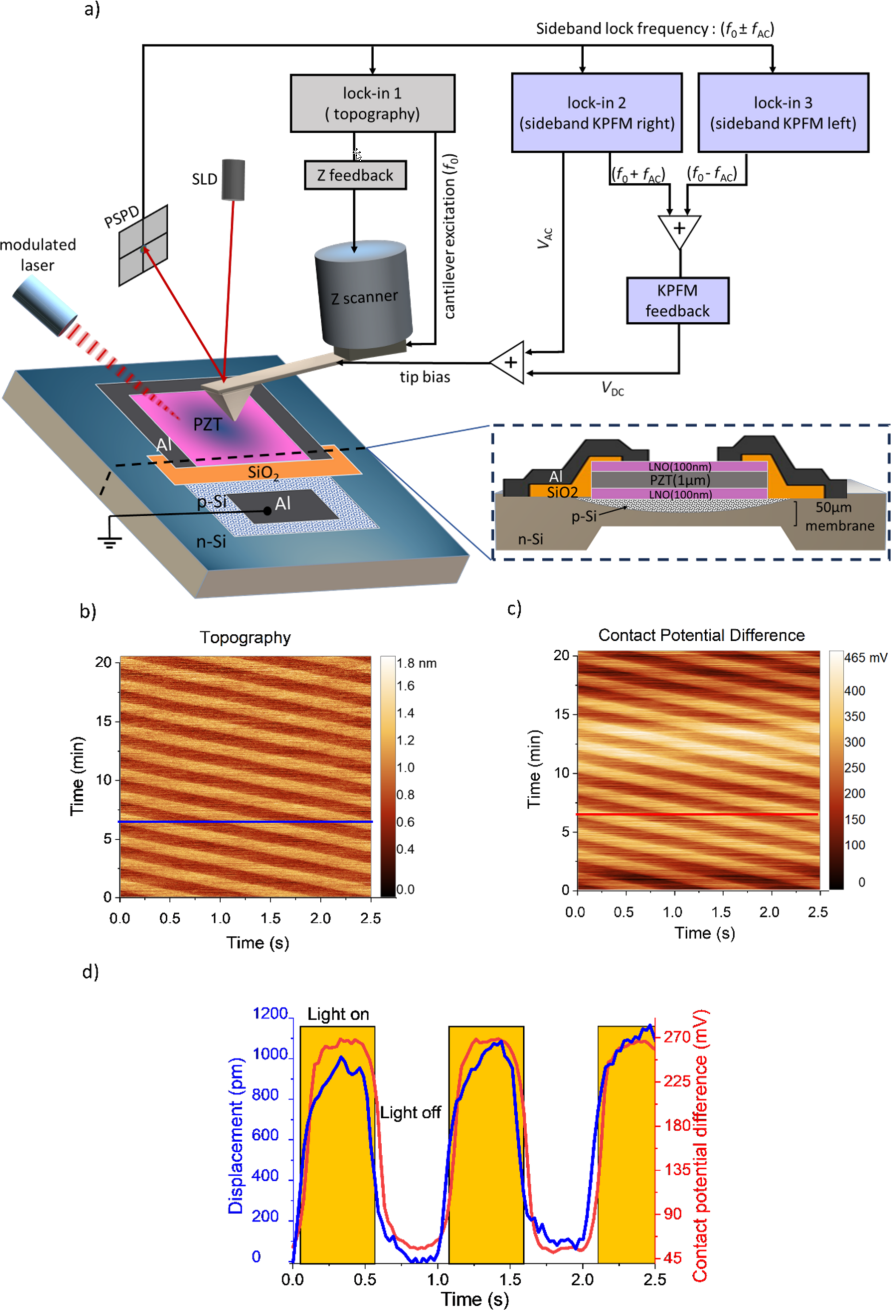
(a) Schematic illustrating the connection diagram of sideband KPFM measuring the piezo/photodiode device under modulated illumination. The top view of the device is shown where the inset represents the cross section of the device stack. (b) Time-dependent topography scan showing height variation under alternating illumination, and (c) corresponding time-dependent CPD under alternating illumination. (d) Temporal behavior of displacement and CPD obtained from averaging multiple linear profiles selected from b and c.

The device type-I employed in this study is a piezo/photodiode device, fabricated following a previously reported procedure [32], where the piezoelectric material, lead zirconate titanate (PZT), was integrated onto a silicon photodiode. The PZT layer was sandwiched between two lanthanum nickelate (LNO) electrodes and ultimately the backside of the silicon substate was etched to enhance the motion of the membrane. More detailed information on the fabrication and cross-sectional scanning electron microscopy images can be found in [32]. The top view of the piezo/photodiode device is given in Figure 1a, where the inset represents the cross section of the device stack. Owing to the integration of PZT with silicon processing and operation at low voltages, this device can be actuated by either modulated illumination or electrical bias.

The device type-II used in this study had the same configuration as the device type-I only without the photodiode. A series of devices with different sizes were fabricated in which the active device dimensions were (7.6 × 7.6), (5.2 × 5.2), (2.8 × 2.8) and (1.4 × 1.2) mm^2^ labeled as A, B, C, and D, respectively. In the process of fabrication, a 100 nm thick layer of LNO as the bottom electrode was first deposited, using pulsed laser deposition (PLD) technique, on a single crystal silicon wafer. Then, an 850 nm lead barium zirconia titanate (PBZT) and a 150 nm LNO as the top electrode were deposited. The wafer was patterned by a standard photolithographic process, starting with the application and patterning of the photoresist mask for defining the device areas. Subsequently, the excess PBZT and LNO were removed by a wet etching process with a diluted BHF:HNO_3_:H_2_O solution and a HCl solution, respectively. To avoid shunting of the device, a SiO_2_ insulating layer was deposited. Patterned aluminum (Al) contacts which connect to the top and bottom electrodes of the PBZT were fabricated through a lift-off process. The fabrication was finalized by etching circular holes from the backside of the wafer to obtain thin membranes. The sizes of these holes were defined by applying and patterning a photoresist on the backside of the wafer, which was then anisotropically etched by deep reactive ion etching (DRIE) using SF_6_, O_2_, and C_4_F_8_ gases. This final step was carried out to minimize the clamping effect of the actuator on the silicon substrate, thus enhancing the movement of the membrane.

### Kelvin probe force microscopy under modulated illumination

In this experiment, we used KPFM with modulated illumination to study device type-I, namely the piezo/photodiode device. The device was illuminated from the top surface using a laser diode at a wavelength of 628 nm. The light source was installed inside the AFM chamber, and the light beam was aligned towards the active area of the device. The illumination source was modulated by a square waveform voltage at an adjustable frequency, operated through a function generator (GWInstek, SFG-1013). Here, the selected modulation frequency was 1 Hz. It should be noted that the active area of the device stack of piezoelectric layer (PZT) and LNO electrodes is transparent and thus the incoming light from the top reached the Si p–n junction and generated electron–hole pairs, building a potential difference across the junction. The generated photovoltage was applied to the piezoelectric capacitor through the photolithographically defined contacts and induced mechanical displacement in the piezoelectric stack. Kelvin probe force microscopy was employed to measure the photoinduced voltage simultaneously with the displacement at the surface of the top LNO electrode as the bottom electrode was grounded. These measurements were performed with a Park Systems NX10 AFM microscope equipped with Pt/Ir-coated silicon probes (ARROW-EFM from nanoworld). Topographical measurements were performed in amplitude-modulated (AM) AFM. The KPFM measurements were carried out in single-pass sideband mode [33], which is a technique that detects electrostatic force gradients. The connection diagram of the sideband KPFM shown in Figure 1a illustrates multiple lock-in amplifiers employed to excite the cantilever both mechanically and electrically at the same time, and to retrieve simultaneously the amplitude and phase of the movement of the cantilever at different frequencies. The cantilever is excited at its mechanical resonance frequency (*f*_0_) executed by the lock-in amplifier 1 and the generated topography signal is controlled by the Z feedback. A sinusoidal AC bias (*V*_AC_) with drive of 1 V and frequency (*f*_AC_) of 5 kHz is applied to the tip through lock-in 2, generating a signal with a frequency of *f*_0_ ± *f*_AC_ near the cantilever resonance. Modulating the tip with *V*_AC_ while the cantilever is oscillating near its resonance frequency leads to frequency mixing and intermodulation of the two frequencies (*f*_0_ ± *f*_AC_) [34]. The lock-in amplifiers 2 and 3 are fed with the vertical deflection signal of the cantilever to measure the sideband signals at *f*_0_ + *f*_AC_ and *f*_0_ − *f*_AC_. Then, their average is used for the KPFM feedback to adjust the DC bias. If *f*_AC_ is chosen to be small enough, such that the sideband peaks are close to *f*_0_, the amplitude of these peaks will be enhanced by the mechanical resonance of the cantilever leading to a better signal-to-noise ratio. The feedback applies a DC bias (*V*_DC_) matching the potential difference between the tip and the sample, which compensates for the electrostatic force. Therefore, the sidebands disappear. The value of *V*_DC_ corresponds to the contact potential difference.

Measurements of modulated topography and CPD were conducted at the center of the membrane, where the AFM tip was positioned at a single point (zero scan size) to avoid any effect of the topography on the results. In conventional AFM images, each pixel represents the value of a specific signal relative to its position within a defined surface region. Herein, the acquired topography and CPD images shown in Figure 1b and Figure 1c were represented in the time domain. Therefore, the horizontal scale of the images indicates the time at which a given pixel has been acquired in one line, while the vertical scale indicates the temporal evolution of this timeline throughout the measurement process. These scans indicate the reproducibility of acquired displacement and photovoltage under modulated illumination at a single point. To analyze them, we extracted 10 line profiles depicted in blue and red for topography and CPD as a function of time, respectively. The average value of the peak-to-peak amplitude was calculated over the collectively selected profiles. The extracted profiles in Figure 1d were fit using a sinusoidal function in which their peak-to-peak amplitude represents the displacement and the photovoltage. The CPD profile in Figure 1d shows a 50 mV baseline in the dark, which can be attributed to the generation of charge carriers due to the stray light from the super luminescent diode (SLD) of the AFM. The wavelength of the SLD beam employed in the measurements is 830 nm, which can be absorbed by the Si photodiode and lead to a nonzero baseline. However, it does not affect the photovoltage as the peak-to-peak potential was calculated. Comparative measurements over a micrometric area of the sample were performed (see Supporting Information File 1). The CPD of a 1 μm^2^ area at the center of the sample was measured in the dark, under continuous and modulated illumination, respectively. The obtained results indicate that the difference between CPD in the dark and under continuous illumination is similar to that under alternating illumination. It is important to note that the acquired photovoltage for the micrometric measurement is comparable to the locally measured photovoltage. However, we were not able to quantify precisely the displacement of the membrane by conventional imaging since a temperature-induced drift occurs under illumination. Therefore, we modified the method in the point scan mode.

The basic principle of our method lies in the acquisition of light-modulated CPD and vertical displacement at a single point on a two-dimensional grid to unveil the 3D motion of the membrane and the corresponding SPV map. To facilitate light alignment on the sample during measurements, we divided the active area of the device (5.6 × 5.6 mm^2^) into four quadrants. Initially, one of the quadrants was subdivided into a grid consisting of 25 points with steps of 0.7 mm in both *x* and *y* directions, where the displacement and SPV of each point were recorded one after another. To ensure that the cantilever or the AFM head does not block the light from reaching the sample, the sample was manually rotated, and the measurement was performed on the second quarter of the device using the same procedure. As all four quadrants of the sample surface were mapped, the points located at the quadrant boundaries were measured twice during each rotation. Therefore, we took the average of the readout signals for each quadrant boundary. Lastly, all the acquired data for displacement and photovoltage of the four quadrants were stitched together and presented in color maps.

### Time-dependent AFM to measure voltage-driven properties

In this experiment, we studied type-II devices (which do not include the photodiode), and the piezoelectric membrane was excited by an external bias. Time-dependent AFM was employed to determine the voltage-induced displacement of the piezoelectric layer. The AFM measurements were carried out in tapping mode, where the tip was located at a single point of the membrane. The external bias with an amplitude of 2.5 V and frequency of 2 Hz in a square waveform provided by the function generator was applied to the top and bottom Al contacts of the piezoelectric device. The height change of a specific point at the center of the membrane was recorded over time for the utilized voltage and frequency. We selected 10 line profiles from the modulated topography image, took the average over the selected lines similarly to the previous case, and fitted these with a sinusoidal function. The peak-to-peak value of the averaged and fitted data represented the displacement of the membrane for a given point. The cantilever was lifted and moved to the next point and the same measurement was performed. By this approach, spatial mapping of the active area was conducted for four piezoelectric actuators of varying sizes, namely A, B, C, and D. Only one-quarter of the devices were mapped, considering their symmetry. The experiment focused on studying the size-dependent displacement of these devices.

### Simulations

**Figure 2:**
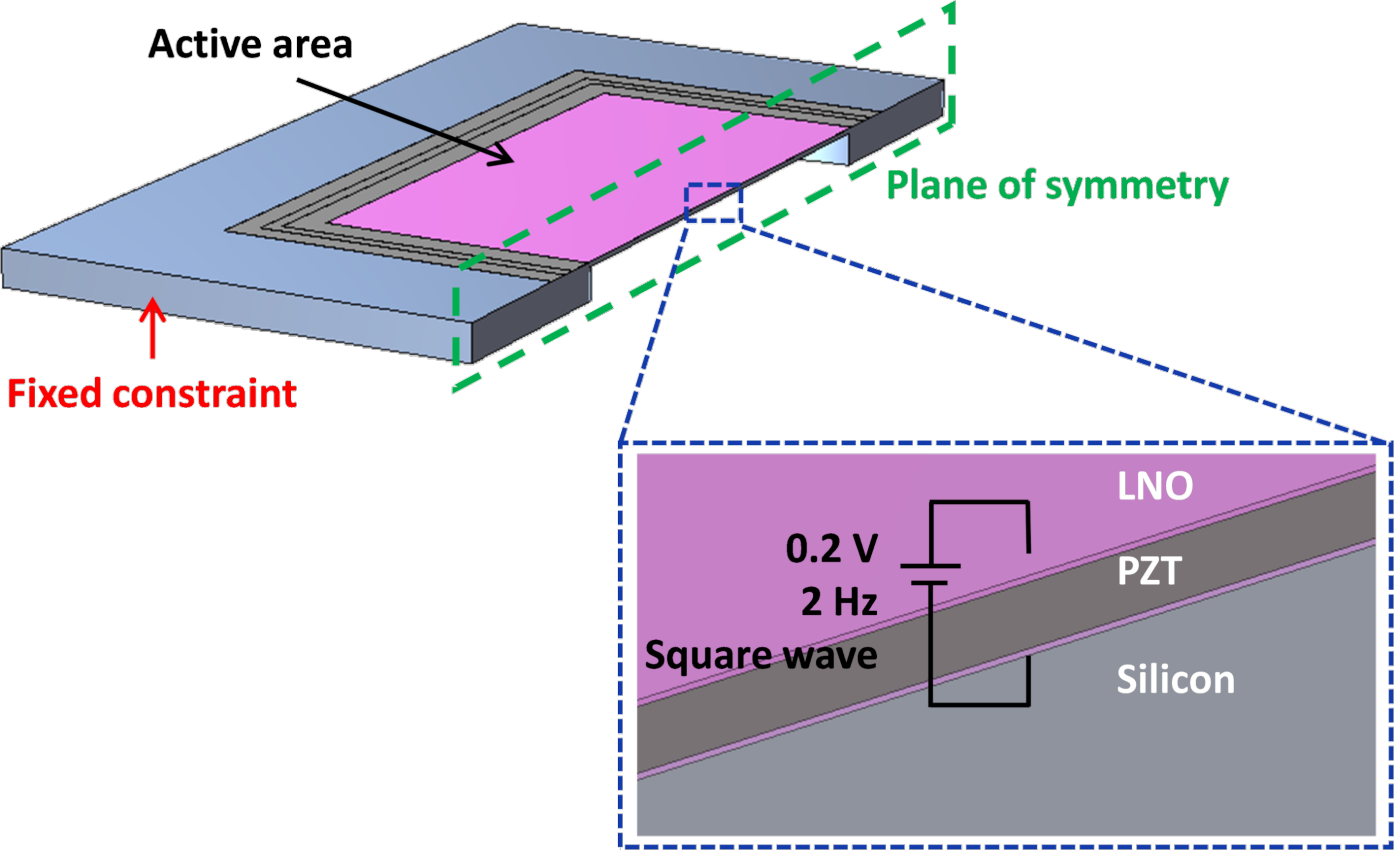
Unit cell of the piezo/photodiode device as simulated in COMSOL.

We used COMSOL Multiphysics for the finite element method (FEM) simulations of our devices. Solid mechanics and electrostatics modules were coupled to facilitate the piezoelectricity calculations. As shown in Figure 2, the symmetry condition is used to reduce the computation cost of the simulations. Analogous to our AFM and KPFM experiments, the sample edges are fixed. Additionally, the effect of gravity on the displacement is taken into account. The voltage excitation signal is introduced to the LNO terminals as shown in the inset of Figure 2.

The material properties of PZT and LNO thin films were derived from the literature and are summarized in Table 1. The strain-charge form is employed for the piezoelectric material.

**Table 1:** Properties of PZT [35–36] and LNO films [37]. Compliance matrix elements (elastic compliance constants, *S*_ij_) and coupling matrix elements (piezoelectric coefficients, *d*_ij_) have the units of 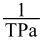 and 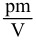, respectively.

Material	Relative permittivity	Poisson’s ratio	Elastic compliance constants					Piezoelectric coefficients		
		
			*S*_11_	*S*_12_	*S*_13_	*S*_33_		*d*_15_	*d*_31_	*d*_33_

PZT	600	0.32	13.8	−4.07	−5.8	20.7		494	−93.5	223
LNO	–∞	0.27								

## Results and Discussion

**Figure 3:**
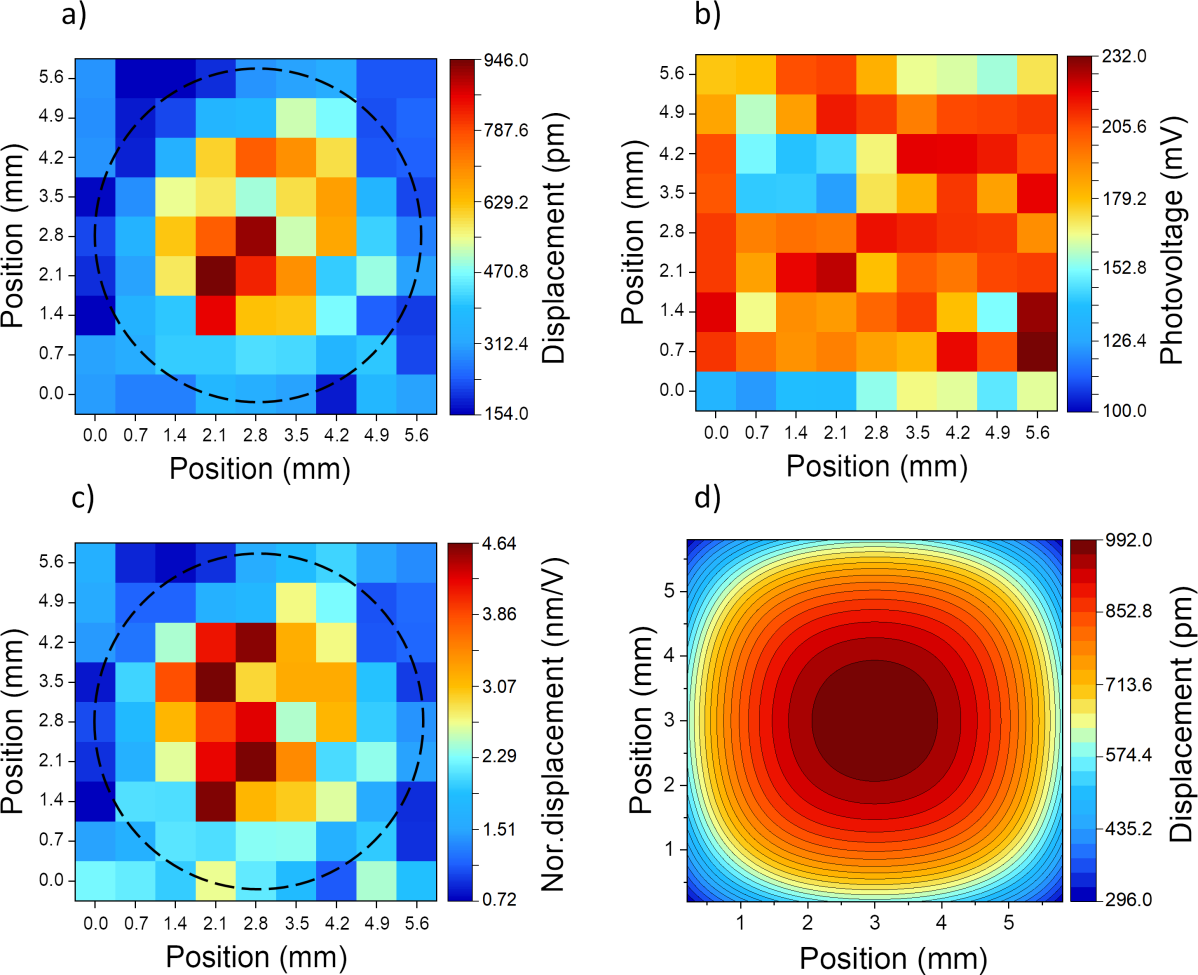
Device type-I corresponding color map of (a) light-induced displacement determined at each point of a predefined grid over the surface of the device. A total of 81 points on the surface active area of 5.6 × 5.6 mm^2^ are mapped. The dashed black circle marks the hole etched on the backside of the silicon substrate. (b) The surface photovoltage of each measured point. (c) Displacement normalized to the corresponding photovoltage at each point. (d) Simulation results of the same sample.

The optoelectronic and mechanical responses of the piezo/photodiode device (device type-I) measured by the KPFM technique are presented in Figure 3. As expected, the maximum displacement is obtained at points positioned at the center of the membrane with a maximum of 946 pm, while decreasing to a few picometers at the side edges. The decay of displacement from the center to the edge is expected from the clamping effect at the edges, where the Si substrate is thicker [38]. The measured CPD shift in Figure 3b indicates that the photovoltage stays nearly constant regardless of the scanned position, and is comparable with the open-circuit voltage (*V*_OC_) of the photodiode (270 mV, measured by a multimeter). By inspecting the displacement and photovoltage color maps it is possible to confirm that the drum-like displacement of the device is due to the flexible center of the membrane and clamped edges, and it is not attributed to the variation of induced photovoltage. The simulation results of the same device (Figure 3d) confirm the shape and range of displacement mapping over the active area. It is instructive to note that ideally the measured photovoltage over the LNO contacts is equal to the *V*_OC_ of the photodiode. However, as can be seen in Figure 3b, this value varies between 100 and 232 mV. This can be attributed to multiple factors, including i) the resistance of the contacts, ii) damages to various points on the top LNO layer from previous measurements or the etching process and hence its lower conductivity, and iii) other factors such as improper light alignment or SPV underestimation by KPFM.

Figure 3c presents the mechanical displacement of the membrane normalized to the photovoltage. These values were obtained by dividing the displacement values for each point in Figure 3a by the corresponding photovoltage in Figure 3b. This analysis confirms that despite fluctuations in the photovoltage, the membrane deformation follows the trend that is expected from simulations. It demonstrates that the maximal displacement that occurred at the center is around 3 to 4.6 nm/V, showing notable performance of the piezo-photodiode device under low photovoltage.

The strength of our approach lies in its capability to decouple the contributions of different aspects of our hybrid system effectively, as the optoelectronic and mechanical properties are simultaneously determined. One of the shortcomings of the implemented KPFM method in our previous work [32] was its operation in dual-pass mode, in which topography measurements were done in the first pass and the CPD in the second pass. Such a measurement can give rise to a phase shift between these two signals.

**Figure 4:**
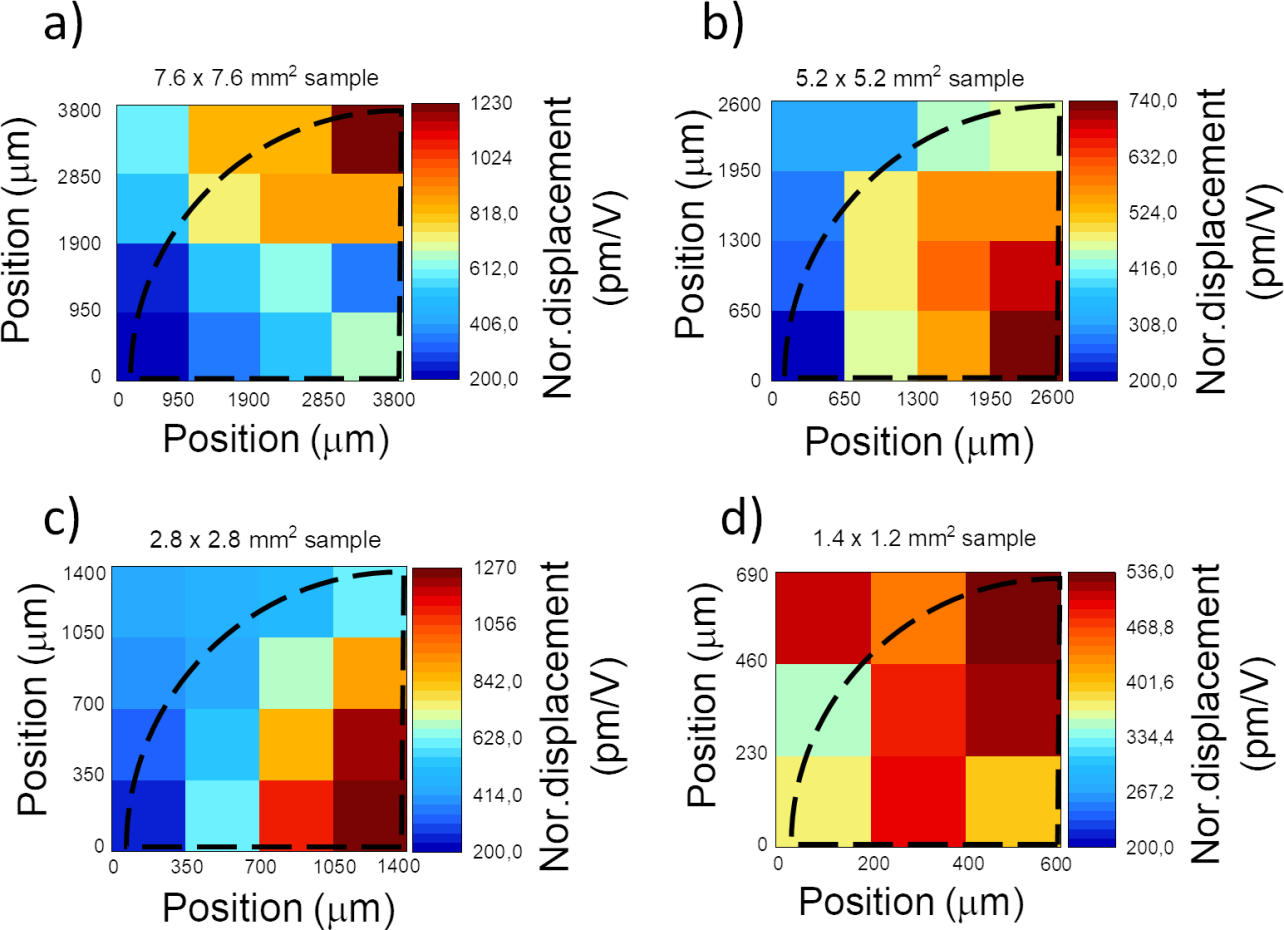
Color map of the quarters of the surface area of device type-II, (a) A with dimensions of (7.6 × 7.6) mm^2^, (b) B with dimensions of (5.2 × 5.2) mm^2^, (c) C with dimensions of (2.8 × 2.8) mm^2^, and (d) D with dimensions of (1.4 × 1.2) mm^2^, where voltage-induced displacement is measured by AFM. The displacement is normalized to the corresponding voltage applied to each point.

Figure 4a–d exhibits the spatial mappings of one-quarter of the voltage-driven piezoelectric actuators (device type-II) with different dimensions from large to small sizes, respectively. The backside etching of the silicon substrate is marked by the dashed line. The measured displacement for each point is normalized to its corresponding applied voltage, shown in picometer per volt (pm/V). It should be noted that the upper limit of the color bars varies according to the maximal displacement value of each device. The maximal displacement is expected at the center of the device, which is the bottom-right corner of each color map. The expected bulging shape is observed for samples B and C shown in Figure 4b and c, respectively. The smallest sample (D) nonetheless shows a relatively uniform displacement, which can arise from the increased stiffness of the membrane as a result of its minuscule dimension (see Figure 4d). Based on the simulations [39], at an excitation voltage of 0.2 V, for active area values larger than ≃6 × 6 mm^2^, the gravitational force dominates the piezoelectric force, causing the membrane to “cave in” in the middle part. This is consistent with the observations in Figure 4. It can also be related to the backside etching of the substrate with poor selectivity, which can form undercuts, causing thinner edges than the central part of the membrane and restricting the movement of the membrane on top.

From the simulations, we expected that the maximum displacement increases with the sample size. However, the experimental results are inconclusive since it can be seen that sample C shows larger displacement than samples A and B despite its smaller size. This may be due to the nonuniform etching of the backside of the sample and thickness variations. While with the presented characterization technique, we cannot fully conclude the reason for the deviation from the expected behavior, the results stress the importance of employing advanced, time-dependent spatial mapping characterization techniques for the development of novel nano/micro-actuators.

## Conclusion

In this study, we demonstrated position-dependent, light-induced measurements of the displacement and photovoltage of a piezo/photodiode device. Kelvin probe force microscopy was employed to record topography and CPD concurrently which allows to distinguish optoelectronic and mechanical responses at the nanoscale. The measured displacements of the light-driven membrane are in good agreement with the simulation results. Moreover, time-dependent AFM was used to investigate position-dependent and size-dependent displacement of piezoelectric actuators driven by an electrical bias. Deviations from the expected membrane behavior stress the importance of advanced characterization methods for the design of novel nano/micro-actuators. The method presented here can offer remarkable opportunities to investigate the properties of devices driven with alternating light or bias voltage, properties of nanoscale mechanical systems, and combinations thereof. As one example, the time-dependent AFM technique in contact mode can be implemented to measure energy conversion efficiency by pressing the AFM tip with a defined counter force against the motion of the system. This allows us to calculate the work that the system is performing against the counter force and with knowledge of the input energy (e.g., absorbed light) the energy conversion efficiency can be determined.

## Supporting Information

File 1 Additional figures.
