# Supplementary material for: Spatial mapping of photovoltage and light-induced displacement of on-chip coupled piezo/photodiodes by Kelvin probe force microscopy under modulated illumination
Source: Beilstein J Nanotechnol. 2023 Nov 6;14:1059–67. doi: 10.3762/bjnano.14.87 (PMC10644008; doi:10.3762/bjnano.14.87)
Supplement: File 1 — Additional figures. [file Beilstein_J_Nanotechnol-14-1059-s001.pdf]

BEILSTEIN JOURNAL OF NANOTECHNOLOGY

Supporting Information

for

Spatial mapping of photovoltage and light-induced displacement of on-chip coupled piezo/photodiodes by Kelvin probe force microscopy under modulated illumination

Zeinab Eftekhari, Nasim Rezaei, Hidde Stokkel, Jian-Yao Zheng, Andrea Cerreta, Ilka Hermes, Minh Nguyen, Guus Rijnders and Rebecca Saive

Beilstein J. Nanotechnol. **2023,** *14,* 1059–1067. doi:10.3762/bjnano.14.87

Additional figures

License and Terms: This is a supporting information file under the terms of the Creative Commons Attribution License (https://creativecommons.org/licenses/by/4.0). Please note that the reuse, redistribution and reproduction in particular requires that the author(s) and source are credited and that individual graphics may be subject to special legal provisions.
The license is subject to the *Beilstein Journal of Nanotechnology* terms and conditions: (https://www.beilstein-journals.org/bjnano/terms)

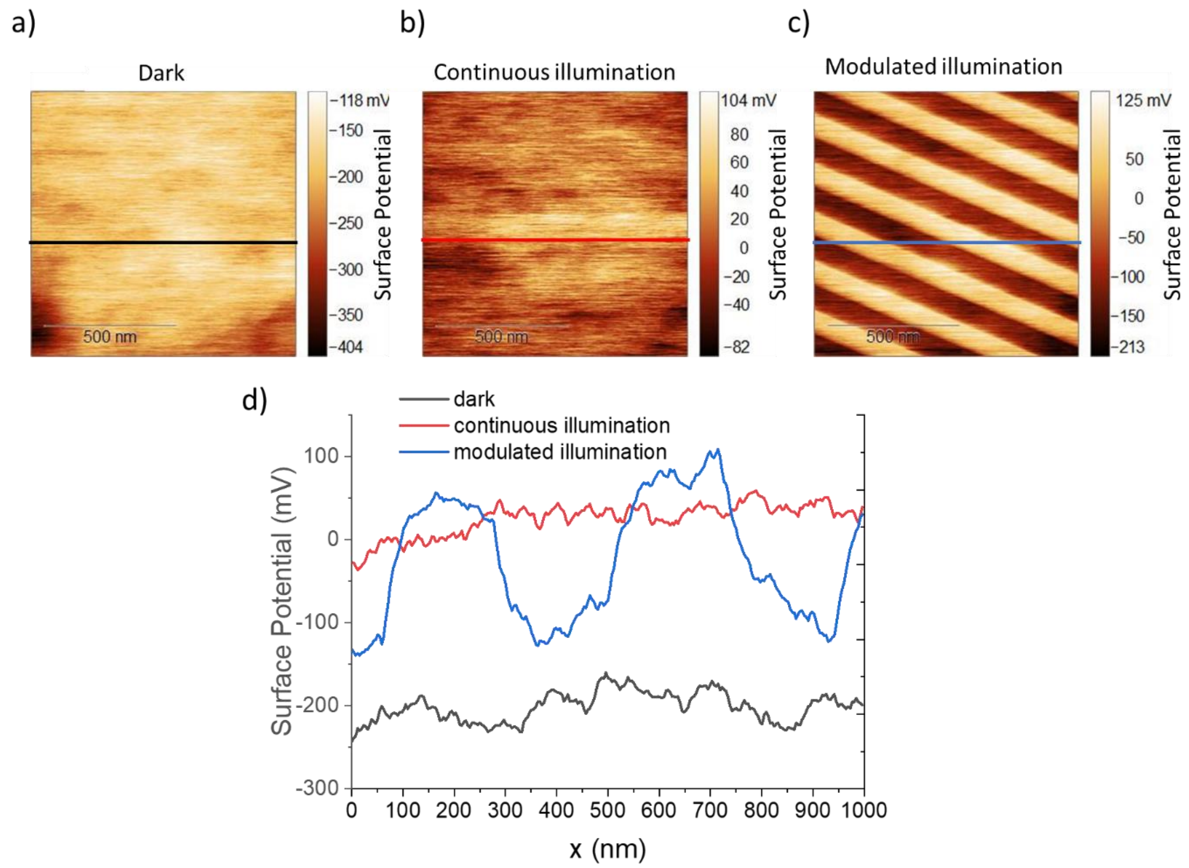

Figure S1: Surface potential of a 1 μm^2 area of the sample measured a) in the dark, b) under continuous illumination, and c) under modulated illumination. d) Black, red, and blue lines show the line profiles of surface potential for dark, continuous and modulated illumination conditions, respectively. The surface potential shift from dark to continuous illumination is comparable to the obtained value under modulated light. The measured surface potential range in the dark and under continuous illumination is quite broad, which can be due to the defects and impurities on the LNO layer. This further explains why modulated illumination is more advantageous compared to conventional continuous illumination.

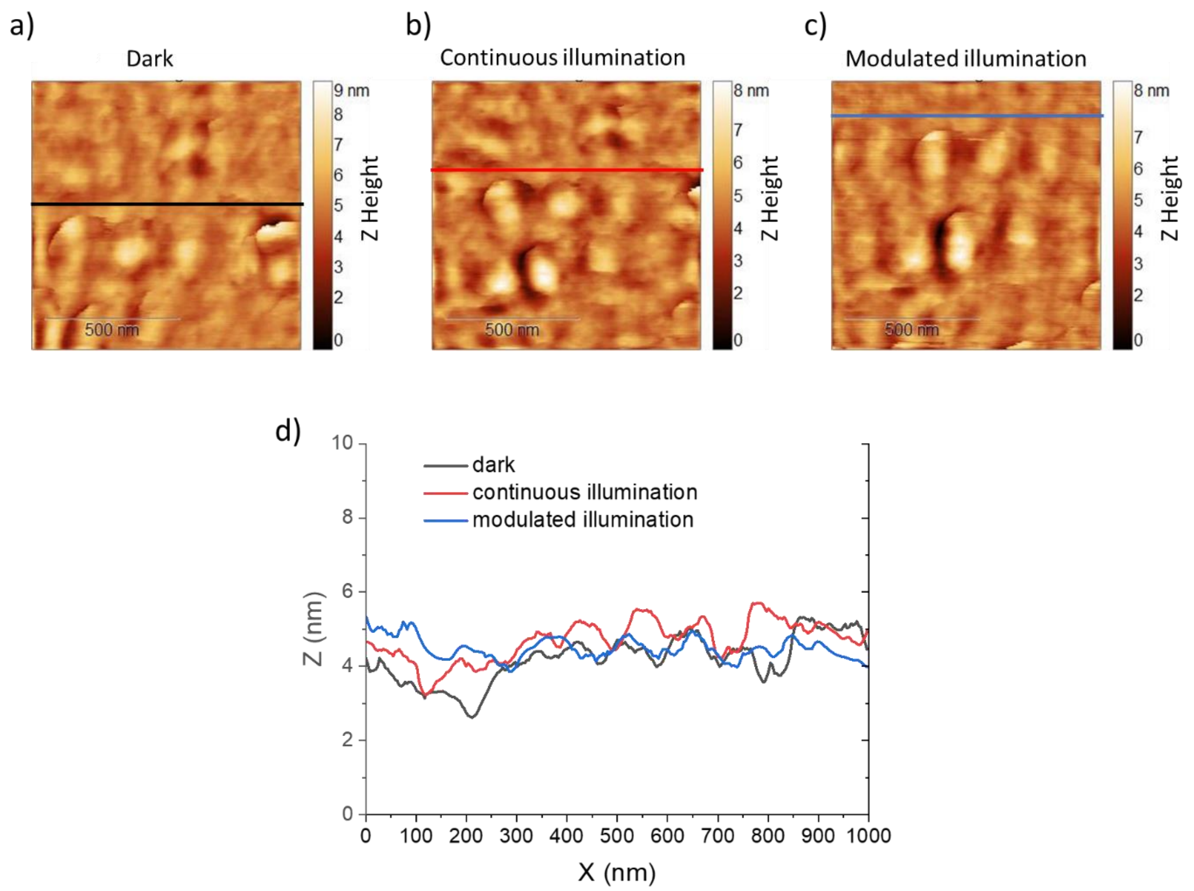

Figure S2: Topographical image of a 1 μm^2 area of the sample measured a) in the dark, b) under continuous illumination, and c) under modulated illumination. d) Black, red, and blue lines show the line profiles of *z* height for dark, continuous, and modulated illumination conditions, respectively. Topography images indicate a noticeable thermal drift that can stem from heat expansion caused by continuous illumination. In addition, the displacement of the membrane cannot be determined from the line profiles of *z* height. Therefore, measurements in single point mode are essential.
